# Poly[μ-aqua-μ_5_-[2-(2,3,6-tri­chloro­phenyl)acetato]-caesium]

**DOI:** 10.1107/S1600536813029395

**Published:** 2013-11-06

**Authors:** Graham Smith

**Affiliations:** a Science and Engineering Faculty, Queensland University of Technology, GPO Box 2434, Brisbane, Queensland 4001, Australia

## Abstract

In the structure of the title complex, [Cs(C_8_H_4_Cl_3_O_2_)(H_2_O)]_*n*_, the caesium salt of the commercial herbicide fenac [(2,3,6-tri­chloro­phen­yl)acetic acid], the irregular eight-coordination about Cs^+^ comprises a bidentate *O:Cl*-chelate inter­action involving a carboxyl­ate-O atom and an *ortho*-related ring-substituted Cl atom, which is also bridging, a triple-bridging carboxyl­ate-O atom and a bridging water mol­ecule. A two-dimensional polymer is generated, lying parallel to (100), within which there are water–carboxyl­ate O—H⋯O hy­dro­gen-bonding inter­actions.

## Related literature

For background information on the herbicide fenac, see: O’Neil (2001 ▶). For the structure of fenac, see: White *et al.* (1979 ▶). For examples of caesium complexes involving coord­inating carbon-bound Cl, see: Levitskaia *et al.* (2000 ▶); Smith (2013 ▶).
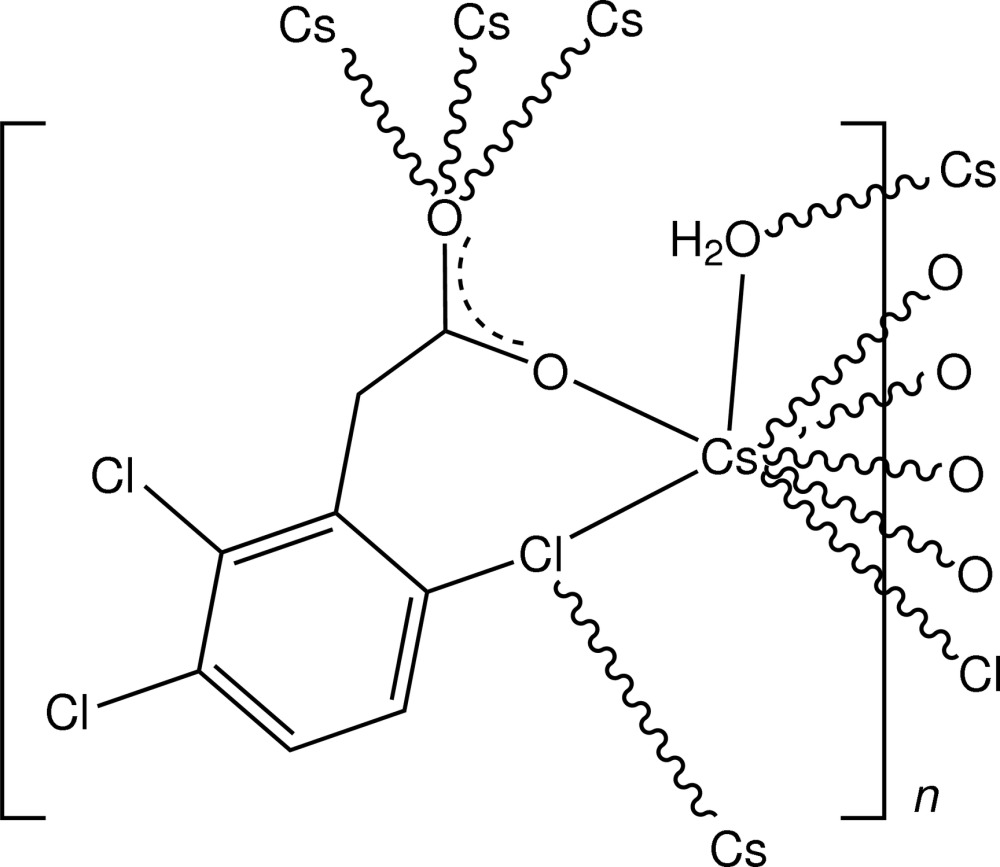


## Experimental

### 

#### Crystal data

[Cs(C_8_H_4_Cl_3_O_2_)(H_2_O)]
*M* *_r_* = 389.39
Monoclinic, 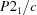

*a* = 17.0606 (12) Å
*b* = 4.9834 (3) Å
*c* = 13.9283 (10) Å
β = 98.127 (6)°
*V* = 1172.29 (14) Å^3^
*Z* = 4
Mo *K*α radiation
μ = 3.82 mm^−1^
*T* = 200 K
0.20 × 0.15 × 0.07 mm

#### Data collection

Oxford Diffraction Gemini-S CCD-detector diffractometer
Absorption correction: multi-scan (*CrysAlis PRO*; Agilent, 2012 ▶) *T*_min_ = 0.582, *T*_max_ = 0.980
7585 measured reflections
2284 independent reflections
1873 reflections with *I* > 2σ(*I*)
*R*_int_ = 0.034

#### Refinement

*R*[*F* ^2^ > 2σ(*F* ^2^)] = 0.050
*wR*(*F* ^2^) = 0.111
*S* = 1.09
2284 reflections
136 parameters
H-atom parameters constrained
Δρ_max_ = 2.18 e Å^−3^
Δρ_min_ = −1.86 e Å^−3^

### 

Data collection: *CrysAlis PRO* (Agilent, 2012 ▶); cell refinement: *CrysAlis PRO*; data reduction: *CrysAlis PRO*; program(s) used to solve structure: *SIR92* (Altomare *et al.*, 1993 ▶); program(s) used to refine structure: *SHELXL97* (Sheldrick, 2008 ▶) within *WinGX* (Farrugia, 2012 ▶); molecular graphics: *PLATON* (Spek, 2009 ▶); software used to prepare material for publication: *PLATON*.

## Supplementary Material

Crystal structure: contains datablock(s) global, I. DOI: 10.1107/S1600536813029395/wm2781sup1.cif

Structure factors: contains datablock(s) I. DOI: 10.1107/S1600536813029395/wm2781Isup2.hkl

Supplementary material file. DOI: 10.1107/S1600536813029395/wm2781Isup3.cml

Additional supplementary materials:  crystallographic information; 3D view; checkCIF report

## Figures and Tables

**Table 1:** Selected bond lengths (Å)

Cs1—Cl6	3.711 (2)
Cs1—O1*W*	3.131 (6)
Cs1—O13	3.246 (7)
Cs1—Cl6^i^	3.646 (2)
Cs1—O1*W* ^i^	3.148 (6)
Cs1—O12^ii^	3.213 (5)
Cs1—O12^iii^	3.103 (6)
Cs1—O12^iv^	3.242 (6)

Symmetry codes: (i) 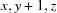; (ii) 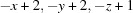; (iii) 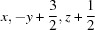; (iv) 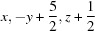.

**Table 2:** Hydrogen-bond geometry (Å, °)

*D*—H⋯*A*	*D*—H	H⋯*A*	*D*⋯*A*	*D*—H⋯*A*
O1*W*—H11*W*⋯O13^ii^	0.97	1.70	2.638 (8)	161
O1*W*—H12*W*⋯O12^v^	0.84	2.40	3.191 (8)	158

Symmetry codes: (ii) 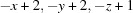; (v) 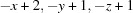.

**Fig. 1.**
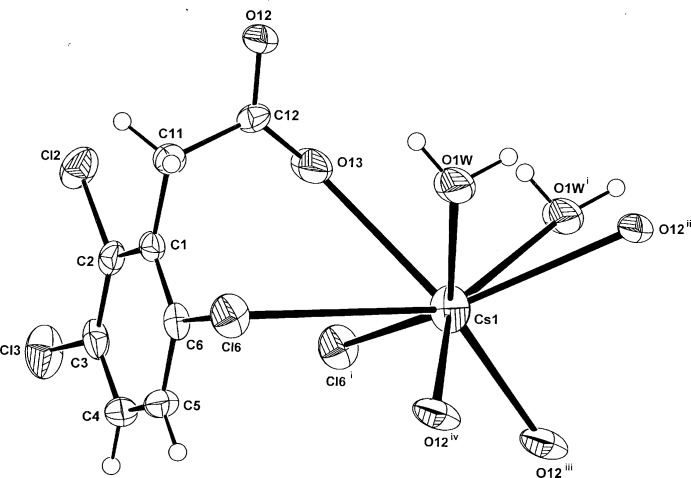
The molecular configuration and atom-numbering scheme for the title compound, with non-H atoms drawn as 40% probability displacement ellipsoids. [For symmetry codes, see Table 1.]

**Fig. 2.**
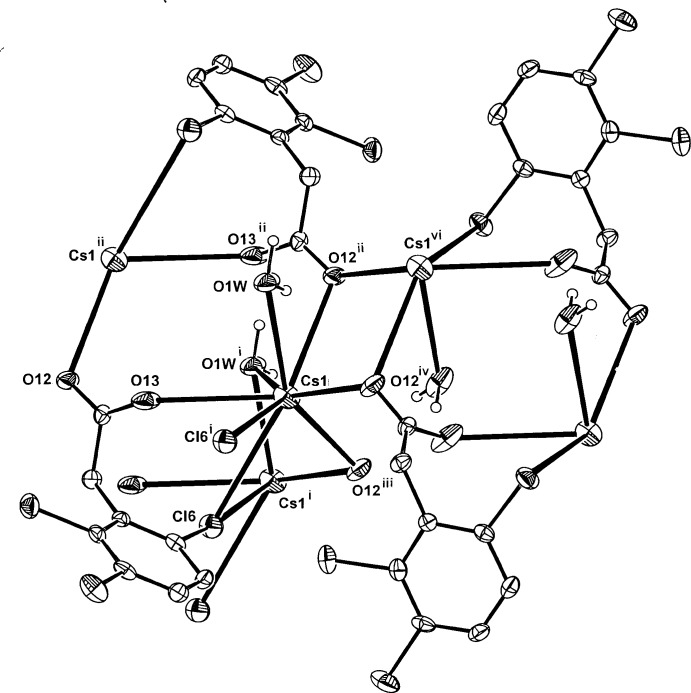
A partial expansion of the Cs+ coordination in the polymer generated by cyclic links through carboxylate, chlorine and water bridges. Ligand H-atoms are omitted. [For symmetry code (vi): -x + 2, y + 1/2, -z + 3/2. For other codes, see Fig. 1 and Table 1.]

**Fig. 3.**
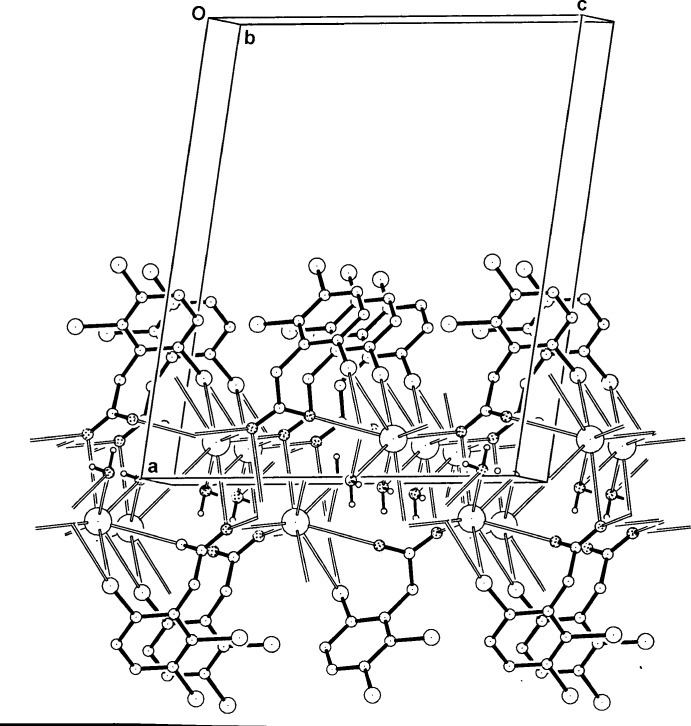
The packing of the sheet structure in the unit cell viewed down b.

## supplementary crystallographic information

### 1. Comment

(2,3,6-Trichlorophenyl)acetic acid (fenac) is a commercial herbicide (O'Neil, 2001) and its crystal structure (White *et al.*, 1979) represents the only entry for this compound in the crystallographic literature. My interest in aromatic carboxylic acid herbicides and in polymeric coordination structures of the alkali metal complexes led to the preparation of the title compound, [Cs(C_8_H_4_Cl_3_O_2_)(H_2_O)]_n_, from the reaction of fenac with caesium hydroxide in aqueous ethanol, and the structure is reported herein.

In this structure (Fig. 1), the irregular eight-coordinate CsClO_7_ polyhedron comprises a bidentate *O:Cl*-chelate interaction involving a carboxylate O-atom (O13) and an *ortho*-related ring substituted Cl-atom (Cl6) which is also bridging, a triple-bridging carboxylate O-atom (O12) and a bridging water molecule O1W (Table 1). A partial expansion of the asymmetric unit in the polymer structure is shown in Fig. 2, forming 4-, 7- and 8-membered cyclic associations linking Cs^+^ ions (a triple bridge involving Cl6, O1W and O12^iii^, extending down *b*). The minimum Cs···Cs^vi^ bridging distance in the structure is 4.4336 (9) Å [for symmetry code (i), see Table 1. For code (vi): -*x* + 2, *y* + 1/2, -*z* + 3/2]. In the Cl bridge, the Cs—Cl bond lengths [3.646 (2) and 3.711 (2) Å] are long compared to those commonly present in the few known examples of caesium complexes having coordinating carbon-bound Cl atoms, *e.g.* 3.46–3.56 Å for a complex in which 1,2-dichloroethane acts as a bidentate chelate ligand (Levitskaia *et al.*, 2000). However, I have previously reported values similar to those in the title complex in the analogous polymeric structure of caesium 4-amino-3,5,6-trichloropyridine-2-carboxylate monohydrate [3.6052 (11)– 3.7151 (11) Å], in which all three ring-substituted Cl-atoms are coordinated (Smith, 2013).

In the crystal structure of the title complex, a polymer with a sheet structure is generated which lies parallel to (100) (Fig. 3), and within which there are _water_O—H···O_carboxylate_ hydrogen-bonding interactions (Table 2).

### 2. Experimental

The title compound was synthesized by heating together under reflux for 10 minutes, 0.5 mmol of (2,3,6-trichlorophenyl)acetic acid and 0.5 mmol of CsOH in 15 ml of 10% ethanol–water. Partial room temperature evaporation of the solution gave thin colourless crystal plates of the title complex from which a specimen was cleaved for the X-ray analysis.

### 3. Refinement

Carbon-bound hydrogen atoms were placed in calculated positions [aromatic C—H = 0.93 Å and methylene C—H = 0.97 Å] and allowed to ride in the refinement, with *U*_iso_(H) = 1.2*U*_eq_(C). Hydrogen atoms of the coordinating water molecule were located in a difference-Fourier synthesis but were subsequently allowed to ride, with *U*_iso_(H) = 1.5*U*_eq_(O). A large maximum residual electron density peak was present (2.176 e^-^ Å^-3^) located at 0.82 Å from Cs1. A short O1W···O1W^ii^ non-bonding contact [2.804 (8) Å] across an inversion centre was also found.

### Crystal data

**Table:**

[Cs(C_8_H_4_Cl_3_O_2_)(H_2_O)]	*F*(000) = 736
*M**_r_* = 389.39	*D*_x_ = 2.206 Mg m^−^^3^
Monoclinic, *P*2_1_/*c*	Mo *K*α radiation, λ = 0.71073 Å
Hall symbol: -P 2ybc	Cell parameters from 2248 reflections
*a* = 17.0606 (12) Å	θ = 3.3–28.0°
*b* = 4.9834 (3) Å	µ = 3.82 mm^−^^1^
*c* = 13.9283 (10) Å	*T* = 200 K
β = 98.127 (6)°	Plate, colourless
*V* = 1172.29 (14) Å^3^	0.20 × 0.15 × 0.07 mm
*Z* = 4	

### Data collection

**Table:**

Oxford Diffraction Gemini-S CCD-detector diffractometer	2284 independent reflections
Radiation source: Enhance (Mo) X-ray source	1873 reflections with *I* > 2σ(*I*)
Graphite monochromator	*R*_int_ = 0.034
Detector resolution: 16.077 pixels mm^-1^	θ_max_ = 26.0°, θ_min_ = 3.4°
ω scans	*h* = −20→21
Absorption correction: multi-scan (*CrysAlis PRO*; Agilent, 2012)	*k* = −6→6
*T*_min_ = 0.582, *T*_max_ = 0.980	*l* = −17→12
7585 measured reflections	

### Refinement

**Table:**

Refinement on *F*^2^	Primary atom site location: structure-invariant direct methods
Least-squares matrix: full	Secondary atom site location: difference Fourier map
*R*[*F*^2^ > 2σ(*F*^2^)] = 0.050	Hydrogen site location: inferred from neighbouring sites
*wR*(*F*^2^) = 0.111	H-atom parameters constrained
*S* = 1.09	*w* = 1/[σ^2^(*F*_o_^2^) + (0.0285*P*)^2^ + 9.056*P*] where *P* = (*F*_o_^2^ + 2*F*_c_^2^)/3
2284 reflections	(Δ/σ)_max_ = 0.001
136 parameters	Δρ_max_ = 2.18 e Å^−^^3^
0 restraints	Δρ_min_ = −1.86 e Å^−^^3^

### Special details

**Table:**

Geometry. Bond distances, angles *etc*. have been calculated using the rounded fractional coordinates. All su's are estimated from the variances of the (full) variance-covariance matrix. The cell e.s.d.'s are taken into account in the estimation of distances, angles and torsion angles
Refinement. Refinement of *F*^2^ against ALL reflections. The weighted *R*-factor *wR* and goodness of fit *S* are based on *F*^2^, conventional *R*-factors *R* are based on *F*, with *F* set to zero for negative *F*^2^. The threshold expression of *F*^2^ > σ(*F*^2^) is used only for calculating *R*-factors(gt) *etc*. and is not relevant to the choice of reflections for refinement. *R*-factors based on *F*^2^ are statistically about twice as large as those based on *F*, and *R*- factors based on ALL data will be even larger.

### Fractional atomic coordinates and isotropic or equivalent isotropic displacement parameters (Å^2^)

**Table:**

	*x*	*y*	*z*	*U*_iso_*/*U*_eq_	
Cs1	0.91683 (3)	1.08611 (9)	0.65098 (4)	0.0524 (2)	
Cl2	0.66412 (12)	1.1809 (4)	0.23490 (12)	0.0501 (6)	
Cl3	0.53476 (12)	1.4225 (4)	0.34892 (17)	0.0616 (8)	
Cl6	0.76993 (11)	0.5765 (4)	0.54801 (14)	0.0508 (6)	
O1W	1.0140 (3)	0.5882 (12)	0.5977 (4)	0.065 (2)	
O12	0.8947 (3)	0.8961 (12)	0.2855 (4)	0.0529 (19)	
O13	0.8658 (3)	1.0892 (13)	0.4175 (5)	0.072 (2)	
C1	0.7124 (3)	0.8850 (12)	0.3931 (4)	0.0274 (17)	
C2	0.6586 (4)	1.0773 (13)	0.3521 (4)	0.0326 (19)	
C3	0.6013 (4)	1.1852 (14)	0.4022 (5)	0.0367 (19)	
C4	0.5961 (4)	1.1051 (15)	0.4948 (5)	0.040 (2)	
C5	0.6479 (4)	0.9137 (15)	0.5385 (5)	0.039 (2)	
C6	0.7052 (4)	0.8101 (13)	0.4877 (5)	0.0322 (19)	
C11	0.7748 (4)	0.7685 (14)	0.3401 (5)	0.036 (2)	
C12	0.8505 (4)	0.9352 (12)	0.3479 (4)	0.0307 (19)	
H4	0.55790	1.17900	0.52840	0.0480*	
H5	0.64430	0.85520	0.60120	0.0470*	
H11A	0.75320	0.75000	0.27210	0.0430*	
H11B	0.78800	0.59030	0.36530	0.0430*	
H11W	1.06400	0.68180	0.60200	0.0970*	
H12W	1.02500	0.45100	0.63200	0.0970*	

### Atomic displacement parameters (Å^2^)

**Table:**

	*U*^11^	*U*^22^	*U*^33^	*U*^12^	*U*^13^	*U*^23^
Cs1	0.0581 (3)	0.0302 (3)	0.0667 (4)	−0.0028 (2)	0.0012 (2)	0.0010 (2)
Cl2	0.0655 (12)	0.0500 (11)	0.0322 (9)	−0.0108 (9)	−0.0020 (8)	0.0071 (8)
Cl3	0.0487 (12)	0.0503 (12)	0.0787 (15)	0.0179 (9)	−0.0157 (10)	−0.0073 (11)
Cl6	0.0477 (11)	0.0492 (11)	0.0530 (11)	0.0041 (9)	−0.0016 (8)	0.0152 (9)
O1W	0.067 (4)	0.073 (4)	0.061 (3)	−0.041 (3)	0.031 (3)	−0.027 (3)
O12	0.039 (3)	0.075 (4)	0.049 (3)	−0.016 (3)	0.021 (2)	−0.026 (3)
O13	0.061 (4)	0.081 (4)	0.083 (4)	−0.042 (3)	0.041 (3)	−0.050 (4)
C1	0.025 (3)	0.026 (3)	0.031 (3)	−0.006 (3)	0.003 (2)	−0.004 (3)
C2	0.035 (4)	0.032 (3)	0.029 (3)	−0.011 (3)	−0.002 (3)	−0.004 (3)
C3	0.022 (3)	0.034 (3)	0.051 (4)	0.003 (3)	−0.006 (3)	−0.011 (3)
C4	0.032 (4)	0.051 (4)	0.039 (4)	−0.001 (3)	0.011 (3)	−0.017 (3)
C5	0.042 (4)	0.047 (4)	0.030 (3)	−0.009 (3)	0.013 (3)	−0.005 (3)
C6	0.025 (3)	0.030 (3)	0.039 (4)	−0.002 (3)	−0.004 (3)	−0.003 (3)
C11	0.035 (4)	0.035 (4)	0.038 (4)	−0.003 (3)	0.010 (3)	−0.010 (3)
C12	0.038 (4)	0.026 (3)	0.029 (3)	0.001 (3)	0.008 (3)	−0.005 (3)

### Geometric parameters (Å, º)

**Table:**

Cs1—Cl6	3.711 (2)	O1W—H12W	0.8400
Cs1—O1W	3.131 (6)	C1—C2	1.392 (9)
Cs1—O13	3.246 (7)	C1—C11	1.496 (9)
Cs1—Cl6^i^	3.646 (2)	C1—C6	1.392 (9)
Cs1—O1W^i^	3.148 (6)	C2—C3	1.387 (9)
Cs1—O12^ii^	3.213 (5)	C3—C4	1.365 (10)
Cs1—O12^iii^	3.103 (6)	C4—C5	1.382 (10)
Cs1—O12^iv^	3.242 (6)	C5—C6	1.385 (10)
Cl2—C2	1.727 (6)	C11—C12	1.527 (10)
Cl3—C3	1.732 (7)	C4—H4	0.9300
Cl6—C6	1.737 (7)	C5—H5	0.9300
O12—C12	1.244 (8)	C11—H11A	0.9700
O13—C12	1.235 (9)	C11—H11B	0.9700
O1W—H11W	0.9700		
			
Cl6—Cs1—O1W	73.58 (10)	Cs1^ii^—O12—Cs1^vi^	89.15 (14)
Cl6—Cs1—O13	62.95 (11)	Cs1^ii^—O12—Cs1^vii^	86.76 (13)
Cl6—Cs1—Cl6^i^	85.27 (4)	Cs1^vi^—O12—Cs1^vii^	103.50 (16)
Cl6—Cs1—O1W^i^	143.35 (11)	Cs1—O13—C12	141.3 (5)
Cl6—Cs1—O12^ii^	136.07 (11)	Cs1—O1W—H12W	126.00
Cl6—Cs1—O12^iii^	64.54 (11)	H11W—O1W—H12W	103.00
Cl6—Cs1—O12^iv^	129.83 (10)	Cs1—O1W—H11W	95.00
O1W—Cs1—O13	80.93 (15)	Cs1^v^—O1W—H11W	149.00
Cl6^i^—Cs1—O1W	142.70 (11)	C2—C1—C11	122.6 (5)
O1W—Cs1—O1W^i^	105.07 (14)	C6—C1—C11	121.8 (5)
O1W—Cs1—O12^ii^	62.90 (14)	C2—C1—C6	115.6 (5)
O1W—Cs1—O12^iii^	69.09 (14)	Cl2—C2—C1	118.2 (5)
O1W—Cs1—O12^iv^	151.22 (14)	Cl2—C2—C3	119.7 (5)
Cl6^i^—Cs1—O13	62.00 (11)	C1—C2—C3	122.1 (5)
O1W^i^—Cs1—O13	80.54 (15)	C2—C3—C4	120.4 (6)
O12^ii^—Cs1—O13	113.08 (14)	Cl3—C3—C4	118.6 (5)
O12^iii^—Cs1—O13	124.78 (15)	Cl3—C3—C2	121.0 (5)
O12^iv^—Cs1—O13	122.59 (15)	C3—C4—C5	119.7 (6)
Cl6^i^—Cs1—O1W^i^	74.34 (10)	C4—C5—C6	119.1 (6)
Cl6^i^—Cs1—O12^ii^	134.05 (11)	Cl6—C6—C5	116.7 (5)
Cl6^i^—Cs1—O12^iii^	128.39 (10)	C1—C6—C5	123.2 (6)
Cl6^i^—Cs1—O12^iv^	64.16 (10)	Cl6—C6—C1	120.2 (5)
O1W^i^—Cs1—O12^ii^	60.21 (14)	C1—C11—C12	114.1 (5)
O1W^i^—Cs1—O12^iii^	150.59 (14)	O12—C12—C11	117.1 (6)
O1W^i^—Cs1—O12^iv^	67.16 (14)	O13—C12—C11	118.5 (6)
O12^ii^—Cs1—O12^iii^	93.30 (14)	O12—C12—O13	124.3 (7)
O12^ii^—Cs1—O12^iv^	90.73 (14)	C3—C4—H4	120.00
O12^iii^—Cs1—O12^iv^	103.50 (15)	C5—C4—H4	120.00
Cs1—Cl6—C6	94.4 (2)	C4—C5—H5	120.00
Cs1—Cl6—Cs1^v^	85.27 (4)	C6—C5—H5	120.00
Cs1^v^—Cl6—C6	173.7 (2)	C1—C11—H11A	109.00
Cs1—O1W—Cs1^v^	105.07 (15)	C1—C11—H11B	109.00
Cs1^ii^—O12—C12	119.0 (4)	C12—C11—H11A	109.00
Cs1^vi^—O12—C12	132.9 (4)	C12—C11—H11B	109.00
Cs1^vii^—O12—C12	114.3 (4)	H11A—C11—H11B	108.00
			
O1W—Cs1—Cl6—C6	−142.6 (3)	O1W—Cs1—O12^iii^—C12^iii^	−172.3 (6)
O1W—Cs1—Cl6—Cs1^v^	31.08 (11)	O13—Cs1—O12^iii^—Cs1^v^	32.8 (2)
O13—Cs1—Cl6—C6	−54.6 (3)	O13—Cs1—O12^iii^—C12^iii^	−110.4 (6)
O13—Cs1—Cl6—Cs1^v^	119.12 (12)	Cl6—Cs1—O12^iv^—Cs1^i^	−112.05 (13)
Cl6^i^—Cs1—Cl6—C6	6.3 (2)	Cl6—Cs1—O12^iv^—Cs1^viii^	159.60 (5)
Cl6^i^—Cs1—Cl6—Cs1^v^	180.00 (5)	Cl6—Cs1—O12^iv^—C12^iv^	39.1 (5)
O1W^i^—Cs1—Cl6—C6	−49.3 (3)	O1W—Cs1—O12^iv^—Cs1^i^	109.0 (3)
O1W^i^—Cs1—Cl6—Cs1^v^	124.40 (17)	O1W—Cs1—O12^iv^—Cs1^viii^	20.7 (3)
O12^ii^—Cs1—Cl6—C6	−150.5 (3)	O1W—Cs1—O12^iv^—C12^iv^	−99.8 (5)
O12^ii^—Cs1—Cl6—Cs1^v^	23.13 (16)	O13—Cs1—O12^iv^—Cs1^i^	−31.8 (2)
O12^iii^—Cs1—Cl6—C6	143.2 (3)	O13—Cs1—O12^iv^—Cs1^viii^	−120.17 (14)
O12^iii^—Cs1—Cl6—Cs1^v^	−43.15 (11)	O13—Cs1—O12^iv^—C12^iv^	119.3 (4)
O12^iv^—Cs1—Cl6—C6	56.6 (3)	Cs1—Cl6—C6—C1	89.9 (5)
O12^iv^—Cs1—Cl6—Cs1^v^	−129.68 (13)	Cs1—Cl6—C6—C5	−90.0 (5)
Cl6—Cs1—O1W—Cs1^v^	−38.11 (11)	Cs1^vi^—O12—C12—O13	153.4 (5)
O13—Cs1—O1W—Cs1^v^	−102.44 (17)	Cs1^vii^—O12—C12—O13	−66.4 (8)
Cl6^i^—Cs1—O1W—Cs1^v^	−96.19 (19)	Cs1^ii^—O12—C12—C11	−142.5 (5)
O1W^i^—Cs1—O1W—Cs1^v^	180.00 (15)	Cs1^vi^—O12—C12—C11	−23.0 (9)
O12^ii^—Cs1—O1W—Cs1^v^	135.7 (2)	Cs1^vii^—O12—C12—C11	117.2 (5)
O12^iii^—Cs1—O1W—Cs1^v^	30.36 (15)	Cs1^ii^—O12—C12—O13	33.9 (9)
O12^iv^—Cs1—O1W—Cs1^v^	110.2 (3)	Cs1—O13—C12—O12	−107.6 (8)
Cl6—Cs1—O13—C12	−39.5 (7)	Cs1—O13—C12—C11	68.8 (9)
O1W—Cs1—O13—C12	36.7 (7)	C6—C1—C2—C3	−0.5 (9)
Cl6^i^—Cs1—O13—C12	−139.1 (7)	C11—C1—C2—Cl2	0.7 (8)
O1W^i^—Cs1—O13—C12	143.7 (7)	C6—C1—C2—Cl2	179.8 (5)
O12^ii^—Cs1—O13—C12	92.0 (7)	C2—C1—C6—Cl6	−178.8 (5)
O12^iii^—Cs1—O13—C12	−19.9 (8)	C2—C1—C6—C5	1.1 (9)
O12^iv^—Cs1—O13—C12	−161.3 (7)	C11—C1—C2—C3	−179.6 (6)
Cl6—Cs1—Cl6^i^—Cs1^i^	180.00 (4)	C11—C1—C6—Cl6	0.3 (9)
O1W—Cs1—Cl6^i^—Cs1^i^	−125.21 (17)	C11—C1—C6—C5	−179.8 (6)
O13—Cs1—Cl6^i^—Cs1^i^	−118.22 (12)	C2—C1—C11—C12	85.3 (7)
Cl6—Cs1—O1W^i^—Cs1^i^	97.39 (19)	C6—C1—C11—C12	−93.7 (7)
O1W—Cs1—O1W^i^—Cs1^i^	179.98 (16)	Cl2—C2—C3—Cl3	0.2 (8)
O13—Cs1—O1W^i^—Cs1^i^	102.15 (17)	Cl2—C2—C3—C4	179.9 (6)
Cl6—Cs1—O12^ii^—Cs1^viii^	−157.48 (7)	C1—C2—C3—Cl3	−179.6 (5)
Cl6—Cs1—O12^ii^—C12^ii^	62.1 (5)	C1—C2—C3—C4	0.2 (10)
O1W—Cs1—O12^ii^—Cs1^viii^	−166.04 (19)	C2—C3—C4—C5	−0.5 (11)
O1W—Cs1—O12^ii^—C12^ii^	53.6 (5)	Cl3—C3—C4—C5	179.2 (6)
O13—Cs1—O12^ii^—Cs1^viii^	128.20 (15)	C3—C4—C5—C6	1.1 (11)
O13—Cs1—O12^ii^—C12^ii^	−12.2 (5)	C4—C5—C6—Cl6	178.4 (6)
Cl6—Cs1—O12^iii^—Cs1^v^	52.02 (11)	C4—C5—C6—C1	−1.5 (11)
Cl6—Cs1—O12^iii^—C12^iii^	−91.1 (6)	C1—C11—C12—O12	−160.0 (6)
O1W—Cs1—O12^iii^—Cs1^v^	−29.16 (14)	C1—C11—C12—O13	23.4 (9)

Symmetry codes: (i) *x*, *y*+1, *z*; (ii) −*x*+2, −*y*+2, −*z*+1; (iii) *x*, −*y*+3/2, *z*+1/2; (iv) *x*, −*y*+5/2, *z*+1/2; (v) *x*, *y*−1, *z*; (vi) *x*, −*y*+3/2, *z*−1/2; (vii) *x*, −*y*+5/2, *z*−1/2; (viii) −*x*+2, *y*+1/2, −*z*+3/2.

### Hydrogen-bond geometry (Å, º)

**Table:**

*D*—H···*A*	*D*—H	H···*A*	*D*···*A*	*D*—H···*A*
O1*W*—H11*W*···O13^ii^	0.97	1.70	2.638 (8)	161
O1*W*—H12*W*···O12^ix^	0.84	2.40	3.191 (8)	158
C11—H11*A*···Cl2	0.97	2.64	3.026 (7)	104
C11—H11*B*···Cl6	0.97	2.61	3.062 (7)	109

Symmetry codes: (ii) −*x*+2, −*y*+2, −*z*+1; (ix) −*x*+2, −*y*+1, −*z*+1.
